# Situation assessment in air combat considering incomplete frame of discernment in the generalized evidence theory

**DOI:** 10.1038/s41598-022-27076-z

**Published:** 2022-12-31

**Authors:** Ying Zhou, Yongchuan Tang, Xiaozhe Zhao

**Affiliations:** 1 School of Electronics and Information, Northwestern Polytechnical University, Xi’an, 710072 Shaanxi China; 2 School of Microelectronics, Northwestern Polytechnical University, Xi’an, 710072 Shaanxi China

**Keywords:** Electrical and electronic engineering, Computer science

## Abstract

For situation assessment in air combat, there may be incomplete information because of new technologies and unknown or uncertain targets and threats. In this paper, an improved method of situation assessment for air combat environment considering incomplete frame of discernment in the evidence theory is proposed to get a more accurate fusion result for decision making in the battlefield environment. First, the situation in air combat is assessed with knowledge. Then, the incomplete frame of discernment in the generalized evidence theory, which is an extension of Dempster–Shafer evidence theory, is adopted to model the incomplete and unknown situation assessment. After that, the generalized combination rule in the generalized evidence theory is adopted for fusion of situations in intelligent air combat. Finally, real-time decision-making in situation assessment can be reached for actions to take. Experiments in situation assessment of air combat with incomplete and uncertain situations show the rationality and effectiveness of the proposed method.

## Introduction

Intelligent information processing has been widely used in the age of internet of things^1–5^. Artificial intelligence technology has brought new weapons to intelligent air combat such as the ALPHA software system^6^. Situation assessment in air combat environment is a key issue for successful defence and attack^7^. However, there are challenges in situation assessment of air combat such as the uncertainty^8^, and the coupled factors^9^. To address these issues, there are many studies focusing on improving the efficiency and accuracy of situation assessment in air combat. Air combat situation assessment often includes threat assessment such as the prediction of enemy’s military intentions. Existing researches mainly include target threat assessment between the two sides basing on the space situation. In^10,11^, the Bayesian networks are used to model the situation assessment environment to get a better understanding of the battlefield scenario. A fuzzy logic-based situation assessment system is developed to help the pilot make the right decision in complex air scenario where there may be multiple friendly aircrafts and/or enemy aircrafts^12^. The situation assessment knowledge of fighter pilots in air combat is applied to model the human situation assessment model in Bayesian networks, which is a new perspective to improve the performance of fighter pilots in air combat^8^. In^13^, the confidence of classification on the air combat data are modelled based on a new algorithm using the naive Bayes theory. The deep neural network is adopted to predict the following situations in air combat environment for improving the probability of winning of unmanned aerial vehicle in air combat^14^. The Dempster–Shafer evidence theory has been applied to model and fuse situation assessments^15,16^, Because of the development of technologies, especially in the background of artificial intelligence, new weapons like the the ALPHA system bring us into a new era of intelligent air combat^17^. New weapon means uncertain, unknown, incomplete and new situation. Information fusion technology is needed to address the uncertain situations including the incomplete and unknown sources.

Dempster–Shafer evidence theory is a typical mathematical tool for information fusion^18,19^. It has been extended as well as been applied in many practical fields such as intelligent decision-making^20–23^, classification^24–26^, clustering^27–29^, supplier selection^30^, and risk analysis^31,32^. Some new theories are proposed for uncertain information modelling and processing based on Dempster–Shafer evidence theory, for example, the transferable belief model^33^, the belief functions^34^, the evidential reasoning theory^35^, the Dezert-Smarandache theory^36^, D Numbers^37^, the belief rule-base model^38,39^, and the complex mass function^40^. To address the potential incomplete information and conflict information fusion because of incomplete frame of discernment, the generalized evidence theory is proposed to model and fuse incomplete and unknown objects^41^ and it is effective in practical applications^42,43^. Thus, this paper choose the generalized evidence theory for modelling and fuse the incomplete situation in the assessment of air combat.

In battlefield environment of air combat, some typical situations under assessment are attack, defence, escape, cruise, reconnaissance, and so on. Because of uncertainty in battlefield environment, one cannot assess all the situations clearly and accurately. For example, a study reports 36 types of maneuvers^14^, you cannot figure out the situation accurately with one sensors or by one expert or one sensor at a time even if the information of two fighters are clearly shown in the space. Information fusion is a must for a real-time decision-making. Thus, a mathematical tool for modelling incomplete situation assessment is needed. Although Dempster–Shafer evidence theory has been applied to model and fuse situation assessments^15,16^, the potential incomplete or unknown situations are ignored. In this paper, the generalized evidence theory is adopted for modelling incomplete situation assessment.

The following sections of this work are organized as follows. In “Preliminaries” section, some basic concepts on the evidence theory and the generalized evidence theory are introduced. In “Situation assessment under incomplete frame of discernment” section, the proposed method for incomplete situation assessment is presented. “Experiment” section gives the experiment and experimental results. “Conclusion” section concludes the work.

## Preliminaries

### Dempster–Shafer evidence theory

Some basic concepts in Dempster–Shafer evidence theory are shown as follows^18,19^.

*Frame of discernment.* The frame of discernment $\Theta$ is a finite nonempty set with mutually exclusive events denoted as ${\Theta\,=\,\left\{ {\theta _1, \theta _2 , \ldots ,\theta _n } \right\} }$, its power set ${2^{\left| \Theta \right| }}$ is defined as follows^18,19^:

1 $$\begin{aligned} {2^{\left| \Theta \right| }} \,=\,\left\{ {\emptyset ,\left\{ {\theta _1 } \right\} ,\left\{ {\theta _2 } \right\} , \ldots ,\left\{ {\theta _n } \right\} ,\left\{ {\theta _1 ,\theta _2 } \right\} , \ldots ,\left\{ {\theta _1 ,\theta _2 \ldots ,\theta _n } \right\} } \right\} . \end{aligned}$$

*Basic probability assignment.* The basic probability assignment (BPA) in Dempster–Shafer evidence theory is defined as a mapping from the power set of ${\Theta }$ to a number between 0 and 1, which satisfies^18,19^:

2 $$\begin{aligned} m\left( \emptyset \right) \,=\,0, 0 \le m\left( A \right) \le 1, \sum \limits _{A \in \Theta } {m\left( A \right) \,=\,1}, \end{aligned}$$

where ${\emptyset }$ is an empty set, *A* is any subsets of ${\Theta }$, the belief function ${m\left( A \right) }$ represents how strongly the evidence supports *A*. The belief assignment ${m\left( \Theta \right) }$ represents the uncertainty of the evidence.

*Dempster’s rule of combination* Dempster’s rule of combination combines two BPAs in a way that the new BPA represents a consensus of the contributing pieces of evidence, it sets intersection putting the emphasis on the common elements of evidence. Dempster’s rule of combination is the orthogonal sum of ${m_1 }$ and ${m_2 }$, denoted by ${\left( {m_1 \oplus m_2 } \right) }$, shown as follows^18^:

3 $$\begin{aligned} m(A)\,=\,\left( {m_1 \oplus m_2 } \right) \left( A \right) {} \,=\,\frac{1}{{1 - k}}\sum \limits _{B \cap C = A} {m_1 (B) \cdot m_2 (C)}, \end{aligned}$$

where *A*, *B*, and *C* are subsets of ${2^{\left| \Theta \right| }}$, *k* is a normalization constant representing the conflict coefficient of two BPAs, *k* is defined as follows^18,19^:

4 $$\begin{aligned} k\,=\,\sum \limits _{B \cap C = \emptyset } {m_1 (B) \cdot m_2 (C)}. \end{aligned}$$

### Generalized evidence theory

In the generalized evidence theory, the BPA in the classical Dempster–Shafer evidence theory is extended and named as the generalized basic probability assignment (GBPA) where the incomplete information is modelled as the mass function of the empty set^41^.

*Generalized combination rule* Give two GBPAs (${m_1}$ and ${m_2}$), the generalized combination rule (GCR) is defined as follows^41^:

5 $$\begin{aligned} \begin{array}{l} m\left( A \right) = \frac{{\left( {1 - m\left( \emptyset \right) } \right) \sum \limits _{B \cap C = A} {{m_1}\left( B \right) \cdot {m_2}\left( C \right) } }}{{1 - K}}, \\ \\ K = \sum \limits _{B \cap C = \emptyset } {{m_1}\left( B \right) \cdot {m_2}\left( C \right) } , \\ \\ m(\emptyset ) = {m_1}\left( \emptyset \right) \cdot {m_2}\left( \emptyset \right) , \\ \\ m(\emptyset ) = \begin{array}{*{20}{c}} 1 &{} {iff} &{} {K = 1} \\ \end{array}. \\ \end{array} \end{aligned}$$

In GCR, the fusion result of two empty sets is defined as ${\emptyset _1} \cap {\emptyset _2} = \emptyset$, which means that the intersection between the two empty sets is still an empty set, which can be considered for further research^44^.

## Situation assessment under incomplete frame of discernment

**Figure 1:**
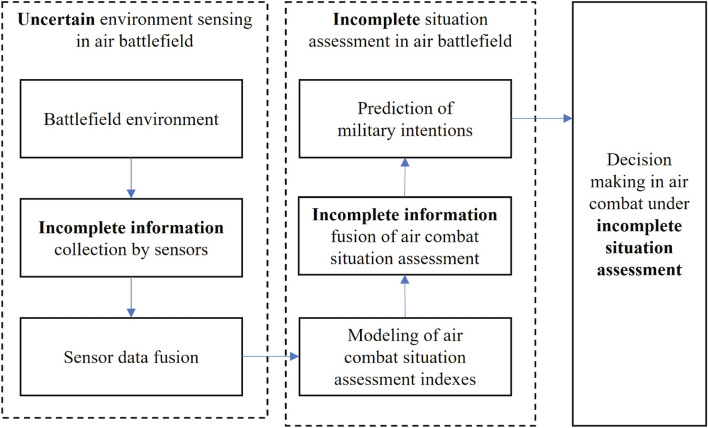
The framework of air combat situation assessment with incomplete situation in air battlefield.

**Figure 2:**
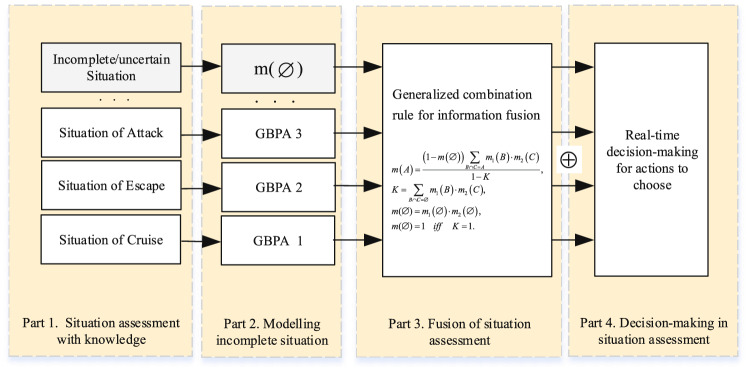
Flow chart of situation assessment considering incomplete information in the evidence theory.

To address the problem of situation assessment in air combat environment with potential incomplete situation, an improved method for situation assessment considering incomplete frame of discernment in the evidence theory is proposed. A typical framework of situation assessment in air combat is presented in Fig. 1, where there are mainly three main processes named uncertain environment sensing in air battlefield, incomplete situation assessment in air battlefield, and decision making in air combat under incomplete situation assessment. The flow chart of the proposed method is shown in Fig. 2.

There are mainly four parts in the proposed method shown in Fig. 2. Firstly, situation assessment with knowledge. Secondly, modelling incomplete situation based on the GBPA including the mass function of the empty set. Thirdly, fusion of situation assessment with the generalized combination rule in the generalized evidence theory. Finally, decision-making of situation assessment based on incomplete information fusion.

### Situation assessment with knowledge

The knowledge of military area should be used to assess the situation in the battlefield environment of air combat. It should be noted that there may be incomplete situation because of the knowledge may not cover all the knew technologies in the enemy. There may be incomplete or unknown situation for both sides in the battlefield environment. The knowledge of the fighter attributes is based on the distance, altitude, speed and angle of the fighter.

### Modelling incomplete situation assessment

In battlefield environment, some typical situations under assessment are attack, defence, escape, and so on. Because of uncertainty in battlefield environment, one cannot assess all the situations clearly and accurately. Thus, a mathematical tool for modelling incomplete situation assessment is needed. In this paper, the generalized evidence theory is adopted for modelling incomplete situation of assessment. For example, an incomplete frame of discernment for situation assessment is $\Omega ={\left\{ {Attack, Cruise, Defence } \right\} }$. Compared to the classical evidence theory, the empty set in the generalized evidence theory is designed for modelling of incomplete information in, Thus, it is an incomplete frame of discernment $\Omega$ in the generalized evidence theory and it can be used to model the unknown situation of assessment.

### Fusion of situation assessment

Situation assessment is based on the information fusion process with the generalized combination rule in the generalized evidence theory. The generalized combination rule is chosen for information fusion after evidence modelling with incomplete situation assessment. The prediction of military intention will be based on the fused situation assessment information. If the evidence modification is based on $(n+1)$ ($n = 1,2,3\ldots$) pieces of evidence, the time of information fusion for the modified evidence is *n*.

6 $$\begin{aligned} m{\left( \cdot \right) _{(0,1,2,\ldots ,n)}} = {\left( {{{\left( {{{\left( {{m_w} \oplus {m_w}} \right) }_1} \oplus {m_w}} \right) }_2} \oplus \cdots } \right) _n}\left( \cdot \right) , \end{aligned}$$

where $\oplus$ means information fusion of the modified evidence ${m_w}\left( \cdot \right)$ is based on Dempster’s rule of combination in Eq. (5). Base on the fusion result, the military intention of the other side can be clearer and it is ready for the final decision-making in situation assessment.

### Decision-making in situation assessment

Real-time decision-making for situation assessment is based on belief degree of fusion results and it decides what action to take for winning the battle. For multiple situations in a proposition, if there is difficult for final decision-making because of a high belief degree on multiple elements in the frame of discernment, then, the pignistic probability transformation in the transferable belief model^33^ can be adopted for assign the belief value to each situation.

## Experiment

The problem description of the experimental setup is adopted from^16^. Two planes of fighters named *r* and *b* are shown in the air combat environment named *OXYZ*. Their distance, azimuth angle, target entry angle and the speed are denoted as *R*, $\alpha$, $\beta$, and *V* respectively. In this circumstance, there may be many types of maneuvers^14^ and it is hard to assess the accurate situation. Two numerical experiments of situation assessment in air combat environment are designed to verify the rationality and effectiveness of the proposed method.

### Experiment 1

Assume that the incomplete frame of discernment for situation assessment is ${\left\{ {Attack, Cruise, Defence } \right\} }$. The situations denoted as GBPAs are given as follows, where the $\emptyset$ means the unclear or unknown situation.

$$\begin{aligned} \begin{array}{l} {m_1}\left\{ {\mathrm{{Attack}}} \right\} = 0.25,{m_1}\left\{ {\mathrm{{Cruise}}} \right\} = 0.25,\\ {m_1}\left\{ {\mathrm{{Defence}}} \right\} = 0.25,{m_1}\left\{ {\mathrm{{\emptyset }}} \right\} = 0.25.\\ {m_2}\left\{ {\mathrm{{Attack}}} \right\} = 0.30,{m_2}\left\{ {\mathrm{{Cruise}}} \right\} = 0.20,\\ {m_2}\left\{ {\mathrm{{Defence}}} \right\} = 0.20,{m_2}\left\{ {\mathrm{{\emptyset }}} \right\} = 0.30.\\ {m_3}\left\{ {\mathrm{{Attack}}} \right\} = 0.40,{m_3}\left\{ {\mathrm{{Cruise}}} \right\} = 0.10,\\ {m_3}\left\{ {\mathrm{{Defence}}} \right\} = 0.20,{m_3}\left\{ {\mathrm{{\emptyset }}} \right\} = 0.30.\\ {m_4}\left\{ {\mathrm{{Attack}}} \right\} = 0.80,{m_4}\left\{ {\mathrm{{Cruise}}} \right\} = 0.05,\\ {m_4}\left\{ {\mathrm{{Defence}}} \right\} = 0.05,{m_4}\left\{ {\mathrm{{\emptyset }}} \right\} = 0.10. \end{array} \end{aligned}$$

With the generalized combination rule in Eq. (5) of the proposed method, the fusion result of GBPAs is as follows. For the first two pieces of evidence, the fusion result is:

$$\begin{aligned} \begin{array}{l} {m_{12}}\left\{ {\mathrm{{Attack}}} \right\} = 0.3964,{m_{12}}\left\{ {\mathrm{{Cruise}}} \right\} = 0.2643,\\ {m_{12}}\left\{ {\mathrm{{Defence}}} \right\} = 0.2643,{m_{12}}\left\{ {\mathrm{{\emptyset }}} \right\} = 0.0750. \end{array} \end{aligned}$$

The results show a not clear situation because the first two pieces of evidence have no high belief value on any of the proposition. The military intention of the other side is not clear.

For the first three pieces of evidence, the fusion result is:

$$\begin{aligned} \begin{array}{l} {m_{123}}\left\{ {\mathrm{{Attack}}} \right\} = 0.6517,{m_{123}}\left\{ {\mathrm{{Cruise}}} \right\} = 0.1086,\\ {m_{123}}\left\{ {\mathrm{{Defence}}} \right\} = 0.2172,{m_{123}}\left\{ {\mathrm{{\emptyset }}} \right\} = 0.0225. \end{array} \end{aligned}$$

After fusion of three pieces of evidence, the belief degree on the situation *Attack* is becoming clearer than two pieces of evidence. The result shows an advantage of using the generalized combination rule.

For all the four pieces of evidence, the fusion result is:

$$\begin{aligned} \begin{array}{l} {m_{1234}}\left\{ {\mathrm{{Attack}}} \right\} = 0.9675,{m_{1234}}\left\{ {\mathrm{{Cruise}}} \right\} = 0.0101,\\ {m_{1234}}\left\{ {\mathrm{{Defence}}} \right\} = 0.0202,{m_{1234}}\left\{ {\mathrm{{\emptyset }}} \right\} = 0.0022. \end{array} \end{aligned}$$

The information fusion results of four pieces of evidence show that there is a high belief degree of 96.75% on the situation of *Attack* based on the four pieces of evidence coming from four sensors or other types of information sources. In this case, maybe a defense situation is needed, follows by an attack action. The real scenery is more complicated than the assumption in the experiment.

### Experiment 2

Assume that, some of the propositions on situation assessments are not based on single situation. It means that the assessment are more uncertain and the belief value of assessment is for more than one situations in a single proposition. For example, the assessment is not sure for a situation that where the enemy is ready for attack or on its cruise under a certain speed, distance, height, angle. Some situation assessments are based on $\left\{ {\mathrm{{Attack}},\mathrm{{Cruise}}} \right\}$ and ${\mathrm{{Attack}},\mathrm{{Defence}}}$ in some pieces of evidence denoted as GBPAs. The GBPAs are given as follows.

$$\begin{aligned} \begin{array}{l} {m_1}\left\{ {\mathrm{{Attack}}} \right\} = 0.25,{m_1}\left\{ {\mathrm{{Attack}},\mathrm{{Cruise}}} \right\} = 0.25,\\ {m_1}\left\{ {\mathrm{{Attack}},\mathrm{{Defence}}} \right\} = 0.25,{m_1}\left\{ {\mathrm{{\emptyset }}} \right\} = 0.25.\\ {m_2}\left\{ {\mathrm{{Attack}}} \right\} = 0.30,{m_2}\left\{ {\mathrm{{Attack}},\mathrm{{Cruise}}} \right\} = 0.20,\\ {m_2}\left\{ {\mathrm{{Attack}},\mathrm{{Defence}}} \right\} = 0.20,{m_2}\left\{ {\mathrm{{\emptyset }}} \right\} = 0.30.\\ {m_3}\left\{ {\mathrm{{Attack}}} \right\} = 0.40,{m_3}\left\{ {\mathrm{{Attack}},\mathrm{{Cruise}}} \right\} = 0.10,\\ {m_3}\left\{ {\mathrm{{Attack}},\mathrm{{Defence}}} \right\} = 0.20,{m_3}\left\{ {\mathrm{{\emptyset }}} \right\} = 0.30.\\ {m_4}\left\{ {\mathrm{{Attack}}} \right\} = 0.80,{m_4}\left\{ {\mathrm{{Attack}},\mathrm{{Cruise}}} \right\} = 0.05,\\ {m_4}\left\{ {\mathrm{{Attack}},\mathrm{{Defence}}} \right\} = 0.05,{m_4}\left\{ {\mathrm{{\emptyset }}} \right\} = 0.10. \end{array} \end{aligned}$$

With the generalized combination rule in Eq. (5) of the proposed method, the fusion result of GBPAs is as follows. For the first two pieces of evidence, the fusion result is:

$$\begin{aligned} \begin{array}{l} {m_{12}}\left\{ {\mathrm{{Attack}}} \right\} = 0.\mathrm{{7488}},{m_{12}}\left\{ {\mathrm{{Attack}},\mathrm{{Cruise}}} \right\} = 0.0881,\\ {m_{12}}\left\{ {\mathrm{{Attack}},\mathrm{{Defence}}} \right\} = 0.0881,{m_{12}}\left\{ {\mathrm{{\emptyset }}} \right\} = 0.0750. \end{array} \end{aligned}$$

For the first three pieces of evidence, the fusion result is:

$$\begin{aligned} \begin{array}{l} {m_{123}}\left\{ {\mathrm{{Attack}}} \right\} = 0.\mathrm{{9376}},{m_{123}}\left\{ {\mathrm{{Attack}},\mathrm{{Cruise}}} \right\} = 0.0133,\\ {m_{123}}\left\{ {\mathrm{{Attack}},\mathrm{{Defence}}} \right\} = 0.0266,{m_{123}}\left\{ {\mathrm{{\emptyset }}} \right\} = 0.0225. \end{array} \end{aligned}$$

For all the four pieces of evidence, the fusion result is:

$$\begin{aligned} \begin{array}{l} {m_{1234}}\left\{ {\mathrm{{Attack}}} \right\} = 0.\mathrm{{9955}},{m_{1234}}\left\{ {\mathrm{{Attack}},\mathrm{{Cruise}}} \right\} = 0.0008,\\ {m_{1234}}\left\{ {\mathrm{{Attack}},\mathrm{{Defence}}} \right\} = 0.00\mathrm{{15}},{m_{1234}}\left\{ {\mathrm{{\emptyset }}} \right\} = 0.0022. \end{array} \end{aligned}$$

The fusion results show that the proposed method can address the uncertain situation assessment in air combat even the assessment is based on multi-situation. The belief value after information fusion with all the four pieces of evidence on the situation of *Attack* is 99.55%, which means the military intention of the enemy is clear and that is attack. A higher belief on the situation *Attack* than the result in Experiment 1 verifies the generalized combination rule inherits the characteristic of Dempster’s rule of combination that a belief convergence will happen on single subset after information fusion, which is good for decision-making in practical applications.

### Discussion and open issues

Dempster–Shafer evidence theory can model and fuse uncertain information in the space of power set of the frame of discernment. However, the incomplete information is out of the frame of discernment. Thus, the generalized evidence theory was adopted in this work to model and fuse the incomplete situation in air combat environment. The mass value of the empty set in the generalized evidence theory is adopted to model the incomplete situation. The fusion result shows that the generalized combination rule of the generalized evidence theory can contribute to a higher belief degree on the right military intention even if there is incomplete situation.

**Figure 3:**
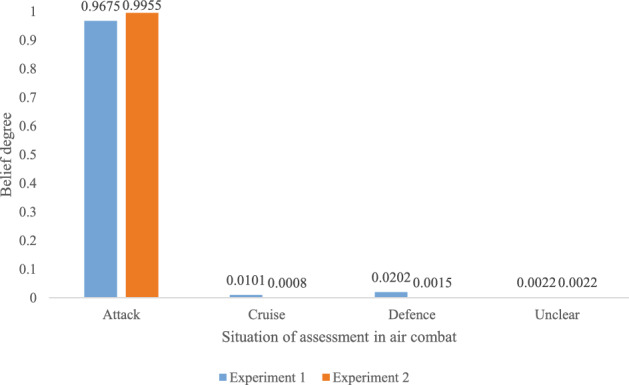
The comparison results between Experiment 1 and Experiment 2 on the situation assessment.

The proposed method inherits the superiority of the generalized evidence theory on modelling incomplete situation. In addition, according to the comparison results in Fig. 3, where the *Unclear* situation means the mass function of the empty set $\emptyset$. It is clear that the proposed method can get a higher belief degree on the GBPA with a single situation, which is good for decision-making in tense air combat environment.

Some open issues need further study. Firstly, the incomplete and uncertain information in the battlefield environment, the acquired information from sensors and the air combat situation understanding according to the military knowledge of many experts can be transmitted to the process of evidence modeling. Secondly, how to measure the uncertain degree in the assessment from sensors and experts needs further work. Thirdly, the current experiment is artificial. How to generate the initial beliefs for the situation assessment information is an open issue related to the topic of generating mass function automatically. In the following research, the algorithms such as the deep learning neural networks^45^, the Markov decision process^46,47^ and hidden Markov model^48,49^ can can be adopted to determine or generate the initial mass functions.

## Conclusion

In this paper, an improved method of situation assessment considering incomplete frame of discernment in the evidence theory is proposed. With an incomplete frame of discernment in the generalized evidence theory, the proposed method can address unknown, uncertain and incomplete situations in assessment of air combat environment. The experimental results verify the effectiveness of the proposed situation assessment method under incomplete situation. It should be noted that the current experiment is very simple and it is only an example of high level information fusion in situation assessment. The following research can focus on more accurate modelling of complex situations and information fusion-based situation assessment.

## Data Availability

All data are included in the manuscript
